# Macrobenthic communities in a shallow normoxia to hypoxia gradient in the Humboldt upwelling ecosystem

**DOI:** 10.1371/journal.pone.0200349

**Published:** 2018-07-17

**Authors:** Maritza Fajardo, Diego Andrade, Jessica Bonicelli, Melanie Bon, Gonzalo Gómez, José M. Riascos, Aldo S. Pacheco

**Affiliations:** 1 Magister en Ecología de Sistemas Acuáticos, Universidad de Antofagasta, Antofagasta, Chile; 2 CENSOR Laboratory, Universidad de Antofagasta, Antofagasta, Chile; 3 Estación Costera de Investigaciones Marinas, Pontificia Universidad Católica de Chile, Santiago de Chile, Chile; 4 Departamento de Oceanografía y Medio Ambiente, Instituto de Fomento Pesquero, Valparaíso, Chile; 5 Programa de Doctorado en Ciencias Aplicadas mención Sistemas Marinos Costeros, Universidad de Antofagasta, Antofagasta, Chile; 6 Estuaries & Mangroves Research group, Universidad del Valle, Cali, Colombia; 7 CENSOR Laboratory, Instituto de Ciencias Naturales Alexander von Humboldt, Universidad de Antofagasta, Antofagasta, Chile; The University of Akron, UNITED STATES

## Abstract

Hypoxia is one of the most important stressors affecting the health conditions of coastal ecosystems. In highly productive ecosystems such as the Humboldt Current ecosystem, the oxygen minimum zone is an important abiotic factor modulating the structure of benthic communities over the continental shelf. Herein, we study soft-bottom macrobenthic communities along a depth gradient–at 10, 20, 30 and 50 m–for two years to understand how hypoxia affects the structure of shallow communities at two sites in Mejillones Bay (23°S) in northern Chile. We test the hypothesis that, during months with shallow hypoxic zones, community structure will be much more dissimilar, thereby depicting a clear structural gradient with depth and correlated abiotic variables (e.g. organic matter, temperature and salinity). Likewise, during conditions of deeper hypoxic zones, communities will be similar among habitats as they could develop structure via succession in conditions with less stress. Throughout the sampling period (October 2015 to October 2017), the water column was hypoxic (from 2 to 0.5ml/l O_2_) most of the time, reaching shallow depths of 20 to 10 m. Only one episode of oxygenation was detected in June 2016, where normoxia (>2ml/l O_2_) reached down to 50 m. The structure of the communities depicted a clear pattern of increasing dissimilarity from shallow normoxic and deep hypoxic habitat. This pattern was persistent throughout time despite the occurrence of an oxygenation episode. Contrasting species abundance and biomass distribution explained the gradient in structure, arguably reflecting variable levels of hypoxia adaptation, i.e. few polychaetes such as *Magelona physilia* and *Paraprionospio pinnata* were only located in low oxygen habitats. The multivariable dispersion of community composition as a proxy of beta diversity decreased significantly with depth, suggesting loss of community structure and variability when transitioning from normoxic to hypoxic conditions. Our results show the presence of semi-permanent shallow hypoxia at Mejillones Bay, constraining diverse and more variable communities at a very shallow depth (10–20 m). These results must be considered in the context of the current decline of dissolved oxygen in most oceans and coastal regions and their impact on seabed biota.

## Introduction

Eastern boundary upwelling ecosystems are characterized for their tremendous amount of primary production and the presence of vast oxygen minimum zones (<2ml/l O_2_) [1]. Oxygen minimum zones in these ecosystems are produced by the combination of two main processes: the presence of cold, nutrient-rich upwelled waters that are naturally poor in dissolved oxygen and the consumption of oxygen via microbial remineralization of organic matter [2]. In the Humboldt Current ecosystem, from the coast of north-central Peru (~4°S) to central Chile (~36°S), the oxygen minimum zone is located over the continental shelf and modifies the structure of seabed communities at shallow and intermediate depths [3, 4]. Communities of macroinvertebrates inhabiting the sediments under the oxygen minimum zone are characterized by low levels of diversity due to a lack of calcifying organisms [5] as well as low biomass [6] but high densities [7, 8]. The latter occurs because few macroinvertebrates are morphologically and physiologically adapted to cope with the oxygen shortage and thus able to flourish under stressful hypoxic conditions [2].

In coastal habitats, such as those found in bays, hypoxic conditions can reach very shallow benthic habitats, amplifying the magnitude of even small increments of this stressor. This can cause a drastic reduction in biodiversity, habitat complexity and resource provision. Monitoring shallow upwelling habitats has become very important since predictions in the context of climate change are that dissolved oxygen is declining in most coastal and oceans basins [9–12] and oxygen minimum zones are vertically expanding in high productivity areas due to an enhancement of upwelling-favorable conditions [13–15]. Indeed, the upper limit of the oxygen minimum zone off the coast of Oregon is located at approximately 600 m deep, but in recent years hypoxic and anoxic episodes have been detected at 50 m with subsequent disappearance of fish and macroscopic benthic invertebrates [16].

Studies addressing the dynamics of benthic communities with regard to the oscillation between normoxic and hypoxic conditions along the Humboldt ecosystem have emphasized the changes occurring during the intrusion of Kelvin waves. These events bring warm, nutrient-poor but well-oxygenated waters onto the continental shelf in otherwise hypoxic habitats during strong El Niño events. During such pulses of oxygenation, species richness, biomass and abundance of macrobenthic communities increases considerably [17, 18] together with switches in the bioturbation capability of the infauna [19]. At decadal-scale, depending on the intensity of the bottom oxygenation, an alternation of dominant biomass states among giant filamentous bacteria (*Beggiatoa* spp.) and meiofauna, polychaete assemblages and giant filamentous bacteria (*Thioploca* spp.) have been described [20]. Also, the temporal variation of the structure of macrobenthic communities in hypoxic conditions seems to be in a cyclical phase with the ongoing decadal cool regimen despite the occurrence of El Niño [21]. However, the consequences of the opposite pattern, which is the intrusion of hypoxic conditions into shallow normoxic soft-bottom habitats, remain largely unknown.

The impacts of hypoxic episodes in shallow bottoms in the Humboldt ecosystem have been described as pulse disturbance [22] in a similar way as habitats with seasonal or unusual strong increments in productivity [23–27]. In central-southern Chile (32–37°S), the upper layer of the oxygen minimum zone impacts benthic communities at the continental shelf break [28, 29]. At this region, after unusually strong upwelling events, hypoxic conditions develop over the shelf, reaching shallow bottoms and causing massive die-off of crustaceans and fish while leaving gastropods unaffected [30]. This may reflect the distinct response and tolerance levels of the benthic organisms to hypoxia [31–33]. This study focuses on shallow benthic communities of northern Chile (23°S), where upwelling is intense year-round. In this region, hypoxic conditions are present at very shallow depths and hypoxia further emerges, depending on monthly or seasonal changes on the upwelling intensity [34]. Thus, the normoxic habitat is compacted and subsequently expands due to the oscillations in the hypoxic conditions. This environmental setting is better described as press disturbance [22] because the effects are not as dramatic as pulse events since it is assumed that benthic organisms experience hypoxia on a regular basis. Herein, we study macrobenthic communities from Mejillones Bay, one of the most intense upwelling areas in northern Chile. From the oxygenation point of view, time series data [34] suggest the existence of a gradient with three bottom habitat types in this bay: (1) a normoxic habitat extending from the surface down to 20 m that does not experience hypoxia any time of the year, (2) a threshold habitat occurring between 20–50 m that is subject to intermittent monthly or seasonal hypoxia and (3) a permanent hypoxic habitat from 50 m down to the lower limit of the oxygen minimum zone.

In the present study, we test the hypothesis that, during months with shallow hypoxic zones, community structure will be much more dissimilar, thereby depicting a clear structural gradient with depth and correlated abiotic variables (e.g. organic matter, temperature and salinity). Likewise, during conditions of deeper hypoxic zones, communities will be similar among habitats as they could develop structure via succession in conditions with less stress.

## Materials and methods

### Study area

**Fig 1:**
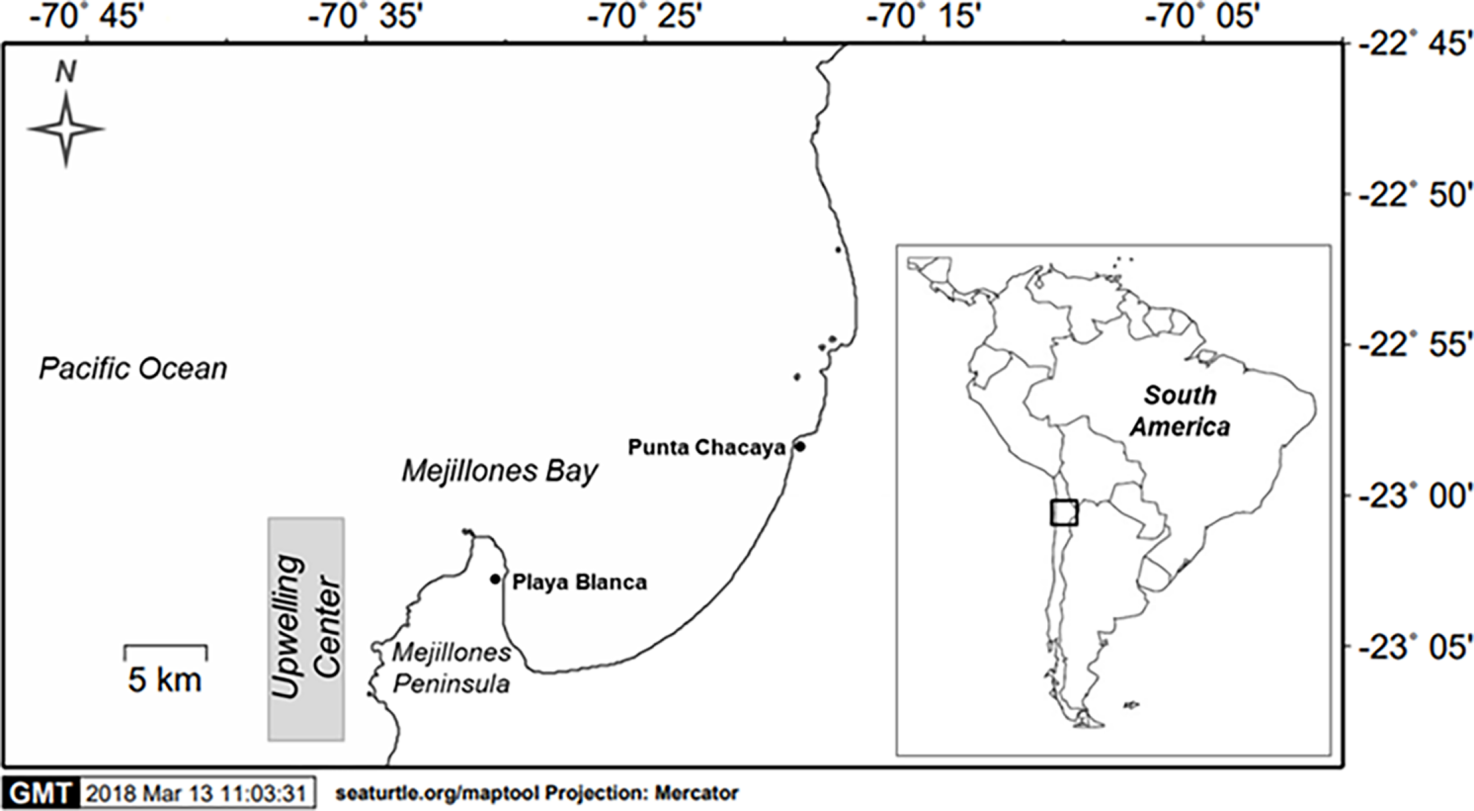
Map of the study site. Location of the two study sites at Mejillones Bay in northern Chile.

Mejillones Bay (23°S) is located at the northern part of Peninsula de Mejillones. Its key characteristic is the high productivity present due to its proximity to an upwelling center in northern Chile (Fig 1). The bay is characterized by cold, upwelled waters that are rich in nutrients as well as a shallow cyclonic flow that enhances water retention inside the bay [35]. Sea surface temperature ranges between 13.5° and 22°C in winter and summer respectively [36]. As previously emphasized, hypoxic waters can reach as shallow as 20 m during seasonal changes as a result of strong upwelling and the circulation pattern [37].

### Sampling strategy

From October 2015 to October 2017, sediment samples were taken monthly at two sites, Punta Chacaya (23° 1’ 4.6”S, 70° 20’ 38.4”W) located at northern extreme of the bay, and Playa Blanca (23° 4’ 18.1”S, 70° 28’ 4.5”W) a site at the inner part of the bay (Fig 1). Sampling was not conducted during March 2016, April and July 2017 due to logistic constrains. Five replicated samples (0.1-m^2^ van Veen grab) were taken at stations located at 10, 20, 30 and 50 m depth at both sites. Sediments were sieved using 500μm mesh screen and the retained biological material (invertebrates and macroalgae) were transferred to plastic bags and fixed with a solution of sea water with 90% ethanol. Sediment subsamples were stored in plastic containers for analysis of both organic matter content and granulometric parameters. At each sampling station, CTDO (Sea Bird 19 plus) vertical hauls were conducted, keeping 50 cm from the bottom. CTDO recorded water column temperature, salinity, dissolved oxygen and density. Since tide variation and boat movement could influence the distance from the boat to the bottom, depths were recorded *a priori* using an echo sounder (GARMIN Echo 200) to ensure the distance above the bottom before CTDO hauls.

### Samples processing

In the laboratory, invertebrates were counted and identified to the lowest taxonomic level possible using a binocular stereoscope, relevant taxonomic literature and the advice of taxonomic experts. Invertebrate and macroalgal biomass was determined by weighing all taxa (mass at 0.01 g precision) after drying at 60°C during 48 h in incubators. Organic matter was calculated as a percentage of weight loss after burning 10 g of sediment at 550°C for 4 h in a muffle furnace.

### Data analysis

Patterns of temporal variability on dissolved oxygen, temperature and salinity were visually examined in “color diagrams” using Ocean Data View software, particularly aiming to detect the sampling months when hypoxic waters reached very shallow depths. Changes in taxonomic richness, abundance and biomass were plotted for an examination of the temporal patterns. Non-metric multidimensional scaling (nMDS) built from the Bray-Curtis (dis)similarity matrix using the abundance for macroinvertebrates and total biomass (macroinvertebrates and macroalgae pooled) data with square root transformation were conducted to visually explore patterns of variation in similarity along the temporal and environmental gradient [38]. Since the grab did not equally take the same amount sediment at all depths, the data was standardized to 100% per samples. Significance for community structure was analyzed using a two-way PERMANOVA [39] considering depth (10, 20, 30 and 50 m), months (sampling months from October 2015 to October 2017) and the interaction term as a fixed factor for each site. Pairwise comparisons for the interaction term “months x depth” for pair levels of the factor depth were examined under the null hypothesis of no significant differences among depths during months with the occurrence of shallow hypoxic conditions. The routine BVSTEP was used to select the subset of species that generates the same multivariate sample pattern, as would the entire community set [40]. This analysis uses Spearman rank correlation to determine the minimum number of variables (i.e. taxa) that show the highest correlation with the complete matrix. This subset of taxa was visualized using nMDS bubble plots to depict variation along the environmental gradient and time. The community composition matrix (presence/absence) using Sørensen distance was used to evaluate the multivariable dispersion (i.e. beta diversity *sensu* [41]) within groups for the factor “months x depth” using a homogeneity test of the multivariate dispersion (PERMDISP [42]). PERMDISP was used to test for similarity in beta diversity among groups (communities at the different depths), testing the null hypothesis of no differences in the multivariate dispersion of the data at the different depths at both sites.

The relationship between the environmental variables and community structure in the gradient was examined using a combination of methods. Principal component analysis (PCA) was conducted on normalized data of the environmental variables (near bottom; temperature, salinity, dissolved oxygen and organic matter content in sediment) to estimate the number of components that provide the major contribution to the environmental variability along the gradient and throughout the study period. In addition, canonical analyses of principal coordinates (CAP) were conducted to find the strongest correlation (canonical correlation) between the community structure matrix and the environmental data set. Finally, to show the relationship between community structure and environmental variables, the highest contributing scores to the percentage of variability of the PCA were correlated with first canonical coordinate scores of the CAP test. All statistical tests were run in PRIMER v7 + PERMANOVA add-on [43].

## Results

### Oceanographic variability and the occurrence of hypoxia

**Fig 2:**
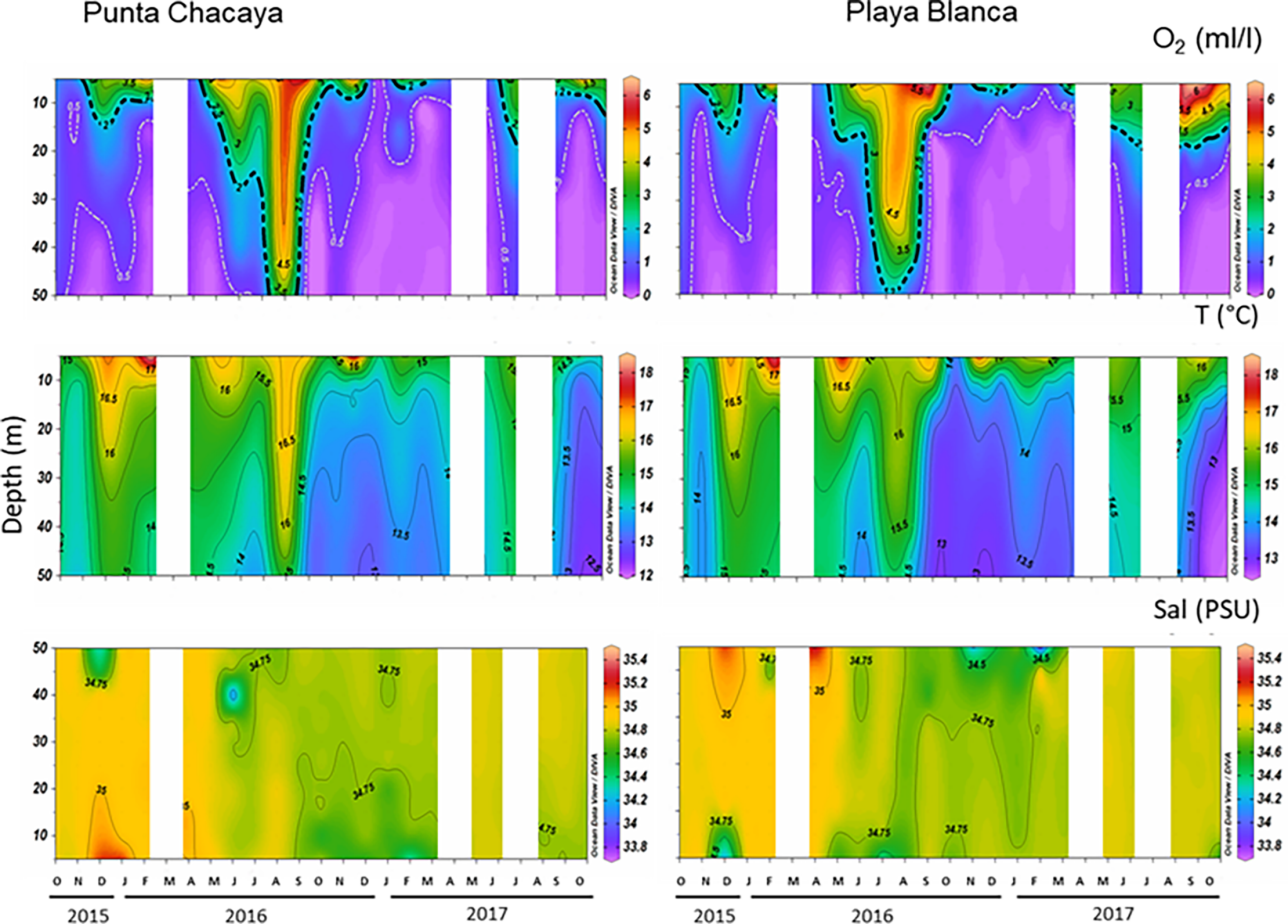
Oceanographic parameters. Time series of temperature of dissolved oxygen, temperature and salinity at during the study period and sites. Blank spaces indicate months with no sampling.

Dissolved oxygen time series from Punta Chacaya and Playa Blanca (Fig 2) show the occurrence of hypoxic water with values below 2 ml/l and 0.5 ml/l. A strong annual signal was observed in the dissolved oxygen time series at both sites. Only during the winter was water column well-oxygenated, presenting oxygen values above 3 ml/l in almost the entire water column. Detectable hypoxia (O_2_ < 2 ml/l) was found only near the bottom at Playa Blanca (Fig 2). During spring and summer, hypoxic water was detected in shallower depths at both sites. Temporal and spatial variability of dissolved oxygen were very similar between sites; however, near the water’s surface, Playa Blanca presented higher oxygen values than Punta Chacaya.

At both ends of the bay, low oxygen is associated with cold water and salinity values around 34.7. During months when the entire water column showed oxygen values lower than 2 ml/l, the water column was cold and presented a weak thermal stratification with salinity values around 34.7. This pattern was mostly observed between September 2016 and March 2017 as well as during September and October 2017 at both sites. During these months, the isoline of 2 ml/l reached up to 10 m depth, with salinity values of approximately 34.7 and temperature values below 14°C in almost the entire water column. Also, interannual differences were observed; the water column throughout spring and into summer of 2015–2016 was warmer and saltier than during the spring and summer of 2016–2017. Unlike temperature and salinity, the oxygen presented less clear interannual differences.

At both sampling sites of the bay, the organic matter content of the sediment increased with depth. Between sites, however, an important difference in the organic matter content present in the sediment was observed. Playa Blanca showed higher organic matter content than Punta Chacaya, especially at 50 m depth. At Punta Chacaya, the percentage of organic matter present in the sediment varied from 1.45±0.6% at 10 m depth to 12.49±3.8% at 50 m depth, and at Playa Blanca it varied from 1.42±1.1% at 10 m depth to 18.12±2.5% at 50 m depth. Table 1 shows the total organic matter content and granulometric parameters at both study sites.

**Table 1:** Values of total organic matter (TOM) content in percentage (mean ± SD) and mean granulometric parameters in phi (Φ) scale (i.e. based on the median and mean of grain size distribution of the sediment), and phi deviation measure (i.e. the degree of sorting) for each sampling station at both sampling sites.

Punta Chacaya				
	10	20	30	50
**TOM**	1.45±0.62	1.48±0.57	2.28±1.74	12.49±3.88
**Mean phi**	medium sand	medium sand	medium fine sand	fine sand
**Sorting**	-1.77±0.52	3.58±0.75	3.42±0.77	2.45±0.28
**Playa Blanca**				
**TOM**	1.42±1.14	1.27±0.46	2.06±1.11	18.12±2.53
**Mean phi**	gross sand	medium sand	medium fine sand	fine sand
**Sorting**	3.29±0.77	0.57±0.52	6.23±0.52	1.15±0.28

### Patterns of taxonomic richness, abundance and biomass

**Fig 3:**
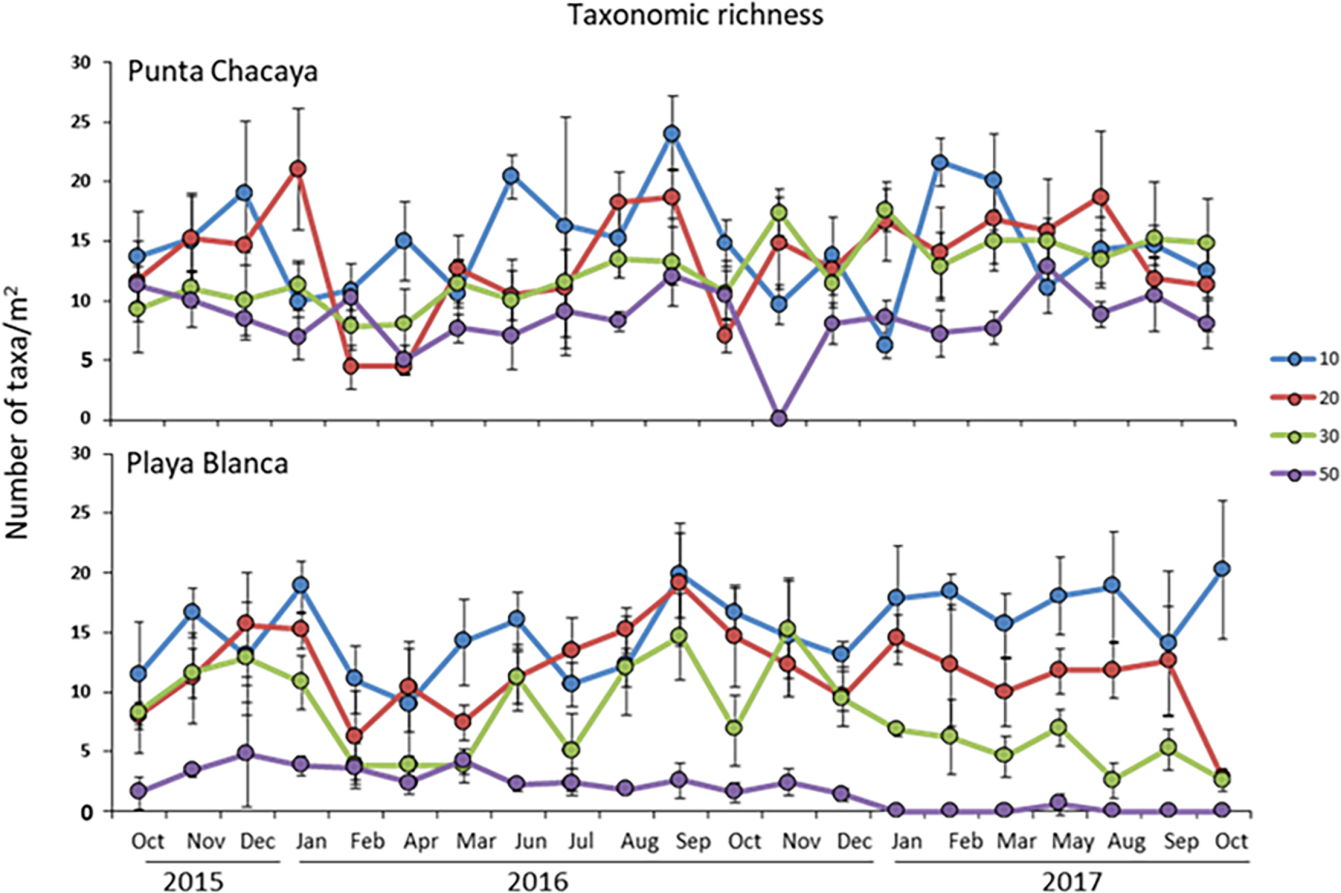
Variability in taxonomic richness. Temporal fluctuations in taxonomic richness (mean ± SD) of the macrobenthos per sampling site during the study period in Mejillones Bay. Color lines represent depth in m.

**Fig 4:**
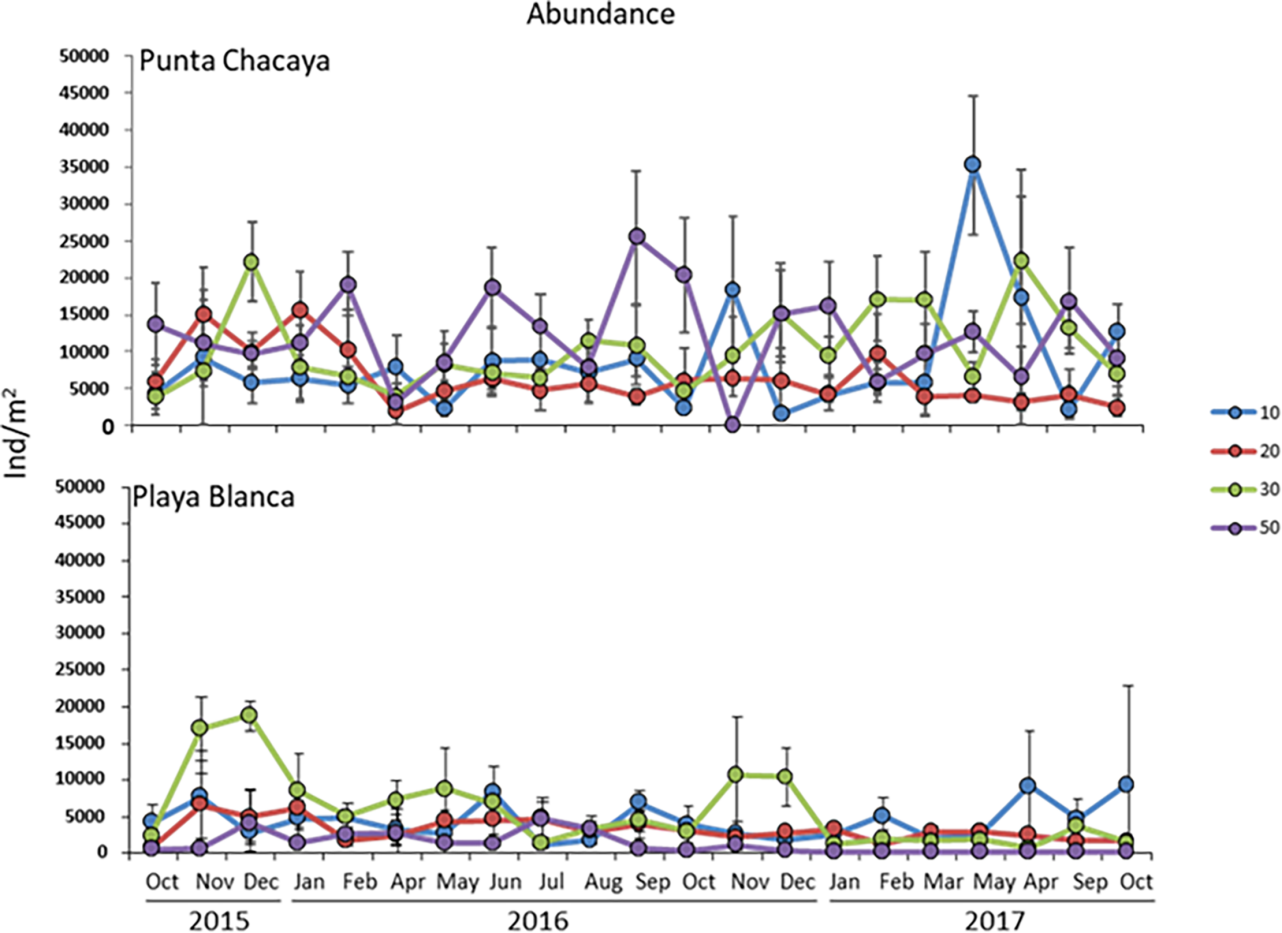
Variability in abundance. Temporal fluctuations in abundance (mean ± SD) of the macrobenthos per sampling site during the study period in Mejillones Bay. Color lines represent depth in m.

**Fig 5:**
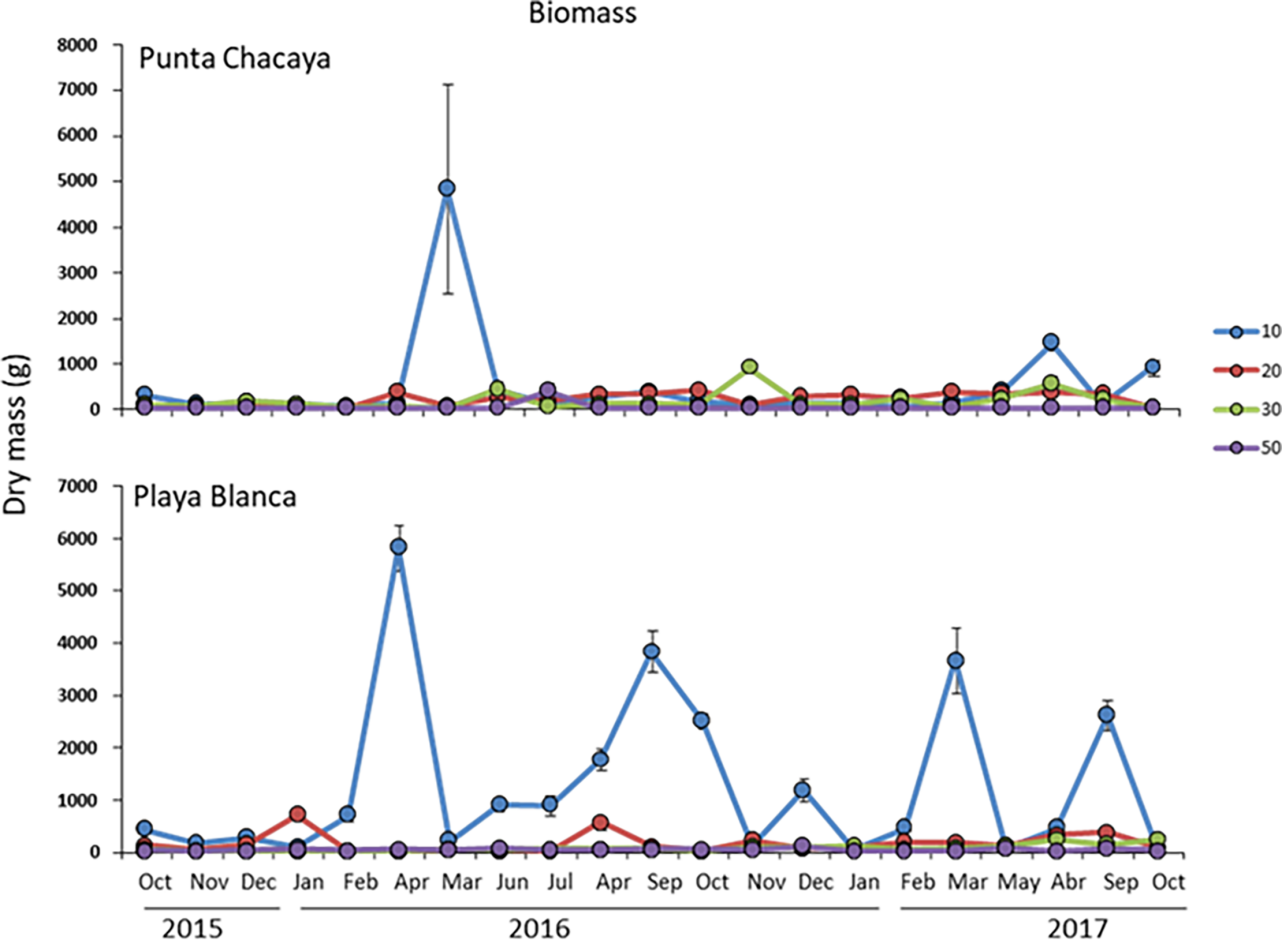
Variability in biomass. Temporal fluctuations in biomass (mean ± SD) of the macrobenthos per sampling site during the study period in Mejillones Bay. Color lines represent depth in m.

A total of 158 taxa (131 at Punta Chacaya and 125 at Playa Blanca) were recorded during the study period, crustaceans being the richest taxa (40%) followed by mollusks (24%) and polychaetes (23%). Nematods, cnidarians, nemerteans, echinoderms, sipunculids and macroalgae contributed a total of 13% of the total number of taxa (S1 Table). In terms of the taxonomical richness, habitats at Punta Chacaya at 10 and 20 m tended to have a higher number of species (though variable throughout time), while habitats at 30 and 50 m tended to have fewer taxa (also variable) (Fig 3). At Playa Blanca, a clear trend of a decreasing number of taxa from shallow to deep habitats exists (Fig 3), though variation between months is present. Patterns in total abundance were also site-specific. At Punta Chacaya, abundance values were similar at 10, 20 and 30 m, but at 50 m values were higher (Fig 4). At Playa Blanca, abundances were similar but with an increase at the 30 m habitat (Fig 4). The most notorious change in the univariate variables between study sites was observed in biomass values. At both Punta Chacaya and Playa Blanca, biomass values were much higher at 10 m depth than at any other depth in the gradient (Fig 5).

### Multivariate patterns of community structure

**Fig 6:**
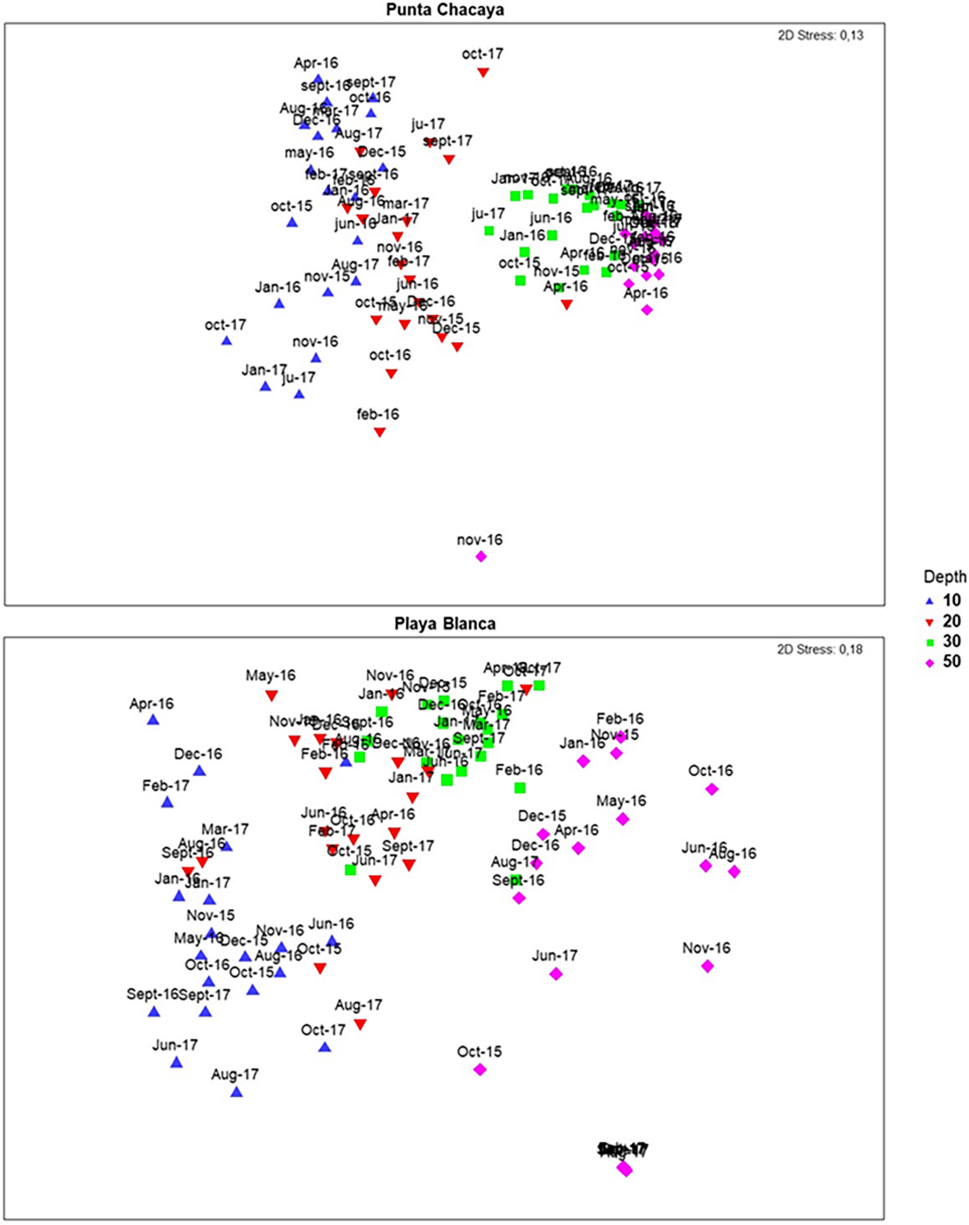
Patterns of variability in community structure. nMDS of the community structure estimated from the abundance data.

**Fig 7:**
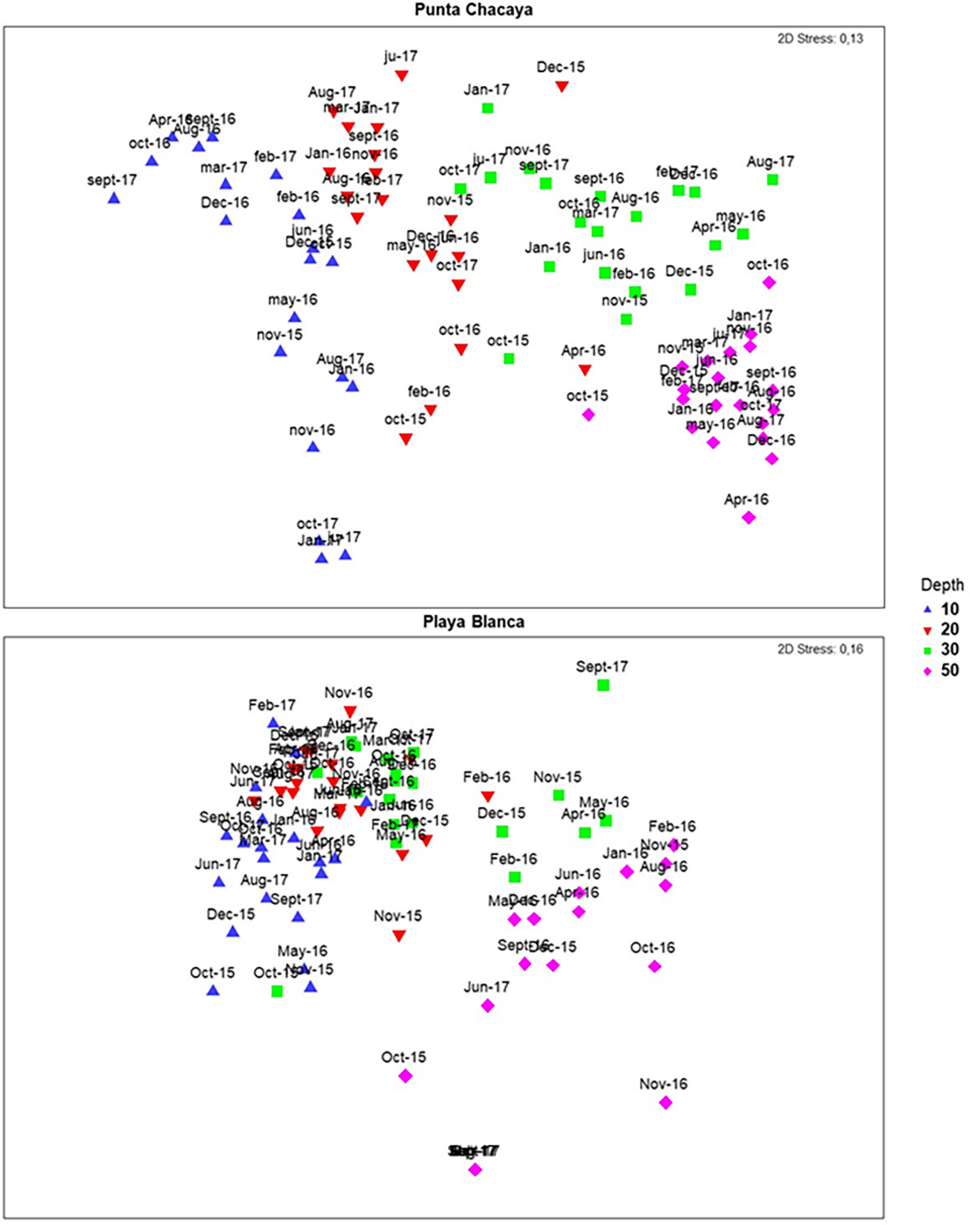
Patterns of variability in community structure. nMDS of the community structure estimated from the biomass data. Symbols are centroids estimated for the depth x month factor.

At both sampling sites, the dissimilarity in community structure among samples increased along the environmental gradient from shallow (10 m) to deeper (50 m) habitats. This pattern is evident for community structure estimates from the abundance and biomass (nMDS plots in Figs 6 and 7) data sets. Although the ordination analysis depicted a clear pattern of dissimilarity along the environmental gradient, some samples become very similar at specific points in time (months). For example, samples from Punta Chacaya at 10 and 20 m are exceptionally similar to one another, yet quite dissimilar from samples at 30 and 50 m, an unusual finding since communities at these depths tend to overlap. A comparable pattern can be observed at Playa Blanca. At Punta Chacaya, there were significant effects for the depth (Pseudo-F_(3, 271)_ = 125.84; P < 0.05), month (Pseudo-F_(16, 271)_ = 10.35; P < 0.05) and the interaction term depth x month (Pseudo-F_(48, 271)_ = 5.28; P < 0.05) (PERMANOVA). All pairwise comparisons for the term depth x month and pair levels of the factor depth were significantly different (all cases P < 0.05). At Playa Blanca, there were significant effects for the same factors, depth (Pseudo-F_(3, 271)_ = 95.15; P < 0.05), month (Pseudo-F_(16, 271)_ = 8.93; P < 0.05) and the interaction term depth x month (Pseudo-F_(48, 271)_ = 5.79; P < 0.05) (PERMANOVA). At this site, no significant effects were detected between 20 and 30 m in December 2016 (t = 1.078; P > 0.05) and again between both depths in March 2017 (t = 1.708; P > 0.05), the rest of the pairwise combinations were significant (all cases P < 0.05).

**Fig 8:**
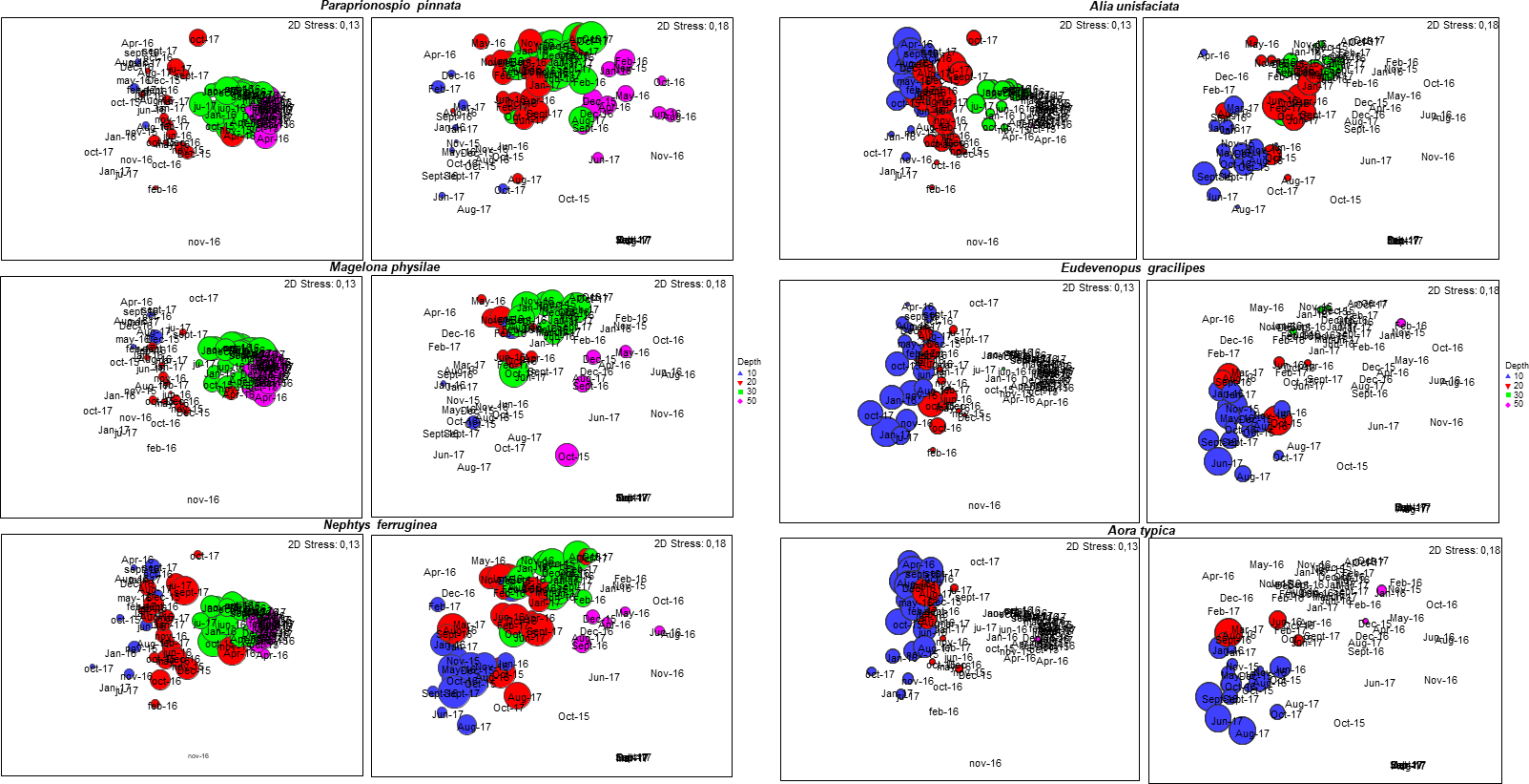
Species contribution to dissimilarity in community structure. nMDS bubble plots showing the contribution of species abundance (selected by the BVSTEP analysis) to the patterns of dissimilarity observed in the environmental gradient and time. The size of the bubble represents the relative abundance of species. Left row contains species for Punta Chacaya and the right contains species for Playa Blanca.

A subset of eight taxa for Punta Chacaya and 23 for Playa Blanca (BVSTEP routine see Table 2 and Fig 8) explained the pattern of similarity for both abundance and biomass throughout time and along the depth gradient. At both sites, some patterns were explained by the same set of species. For example, polychaetes such as *Paraprionospio pinnata* and *Magelona physilae* were abundant at 20, 30 and 50 m hypoxic habitats, while species such as amphipods *Eudevenopus gracilipes* and *Aora typica* were abundant only at 10 and 20 m and nearly absent at 30 and 50 m (Fig 8).

**Table 2:** Sub-set of taxa selected using the BVSTEP analysis. Taxonomic affiliation: (G) gastropod, (B) bivalve, (A) amphipod, (C) crustacean, (O), ostracoda, (P) polychaetes, (Am) amphioxus.

Taxa		Punta Chacaya (ρ = 0,955)	Playa Blanca (ρ = 0,944)
*Nassarius coppingeri*	(G)		X
*Nassarius gayi*	(G)		X
*Alia unifasciata*	(G)	X	X
*Nuculana cuneata*	(B)		X
*Mysella* sp.	(B)		X
*Eudovenopus gracilipes*	(A)	X	X
*Aora typica*	(A)	X	X
*Pinnixia transversalis*	(C)		X
*Pinnixia valdiviensis*	(C)		X
Ostracoda n.n.	(O)		X
*Caprella* sp	(C)		X
*Capitellidae*	(P)		X
*Diopatra chilensis*	(P)		X
*Prionospio ehlersi*	(P)		X
*Prionospio orensanzi*	(P)	X	X
*Cirratullidae*	(P)	X	X
*Paraprionospio pinnata*	(P)	X	X
*Magelona phyllisae*	(P)	X	X
*Nephtys ferruginea*	(P)	X	X
*Maldanidae*	(P)		X
*Glicera* sp.	(P)		X
*Leitoscoloplos chilensis*	(P)		X
*Branchiostoma elongatum*	(Am)		X

Overall, the multivariable dispersion decreased in the transition from shallow normoxic to deep hypoxic habitats. To illustrate, at Punta Chacaya, the average distance to the centroids was 46.97 at 10 m, decreasing to 27.2 at 50 m (Table 3). This reduction of the multivariable dispersion can be also noted on the nMDS plots. Large, spread sample points are notorious at 10 m while samples at 50 m are displayed as a tight cluster (Fig 6). In line with these results, there were significant effects for Punta Chacaya (F = 176.6, df_1_ = 3, df_2_ = 416, P < 0.05) and Playa Blanca (F = 40.694, df_1_ = 3, df_2_ = 415, P < 0.05) (PERMDISP test).

**Table 3:** PERMDISP results, Sørensen distance to centroid (means and standard errors).

Punta Chacaya		
Depth	Average	SE
**10**	46.9	0.68
**20**	45.4	0.66
**30**	33.3	0.77
**50**	27.2	0.74
**Playa Blanca**		
**10**	44.9	0.66
**20**	41.8	0.73
**30**	38.3	0.76
**50**	33.7	0.85

### Community structure and the environmental gradient

**Fig 9:**
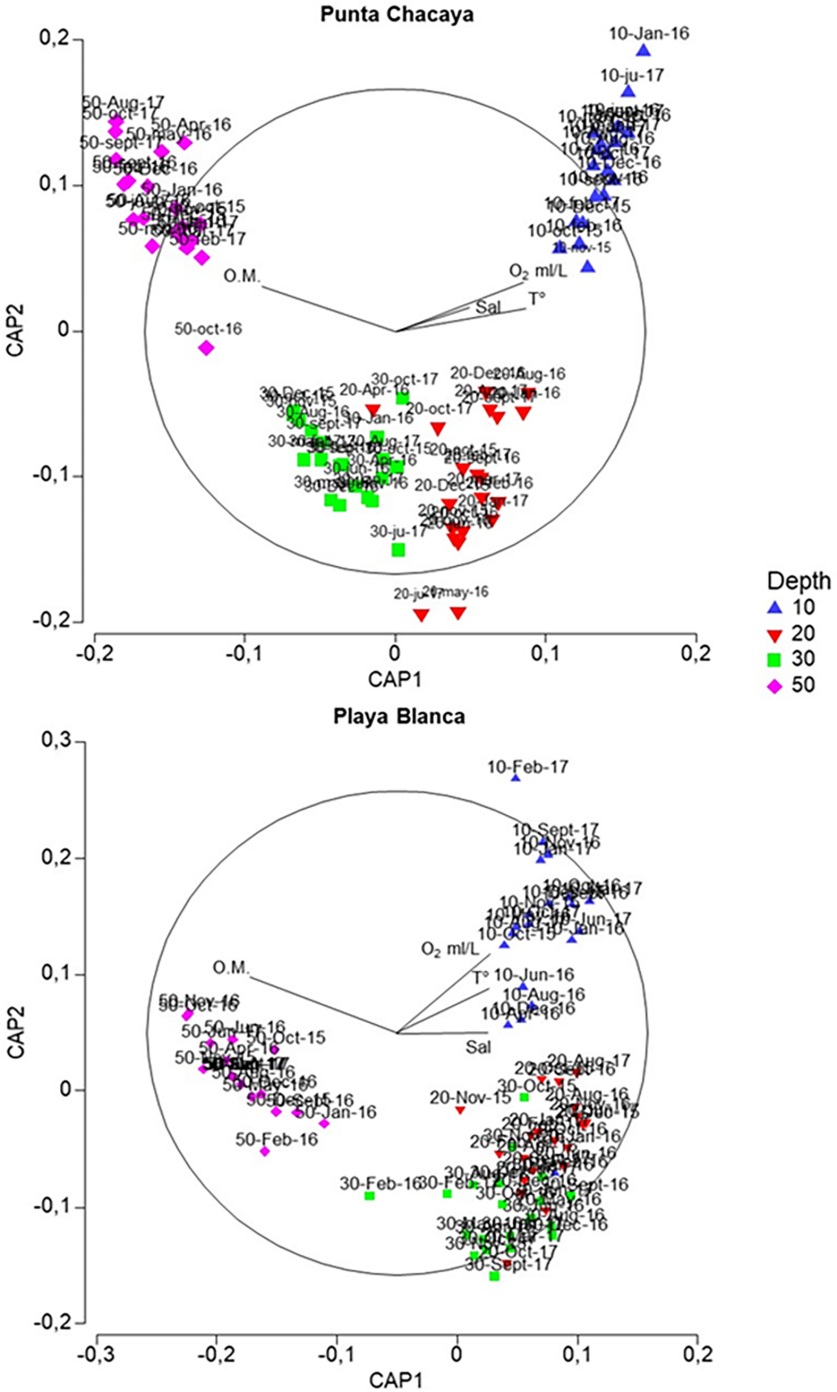
Relationships between community and environmental parameters. CAP ordination plots used as a discriminant function to show main grouping of community structure in function to the environmental parameters.

**Fig 10:**
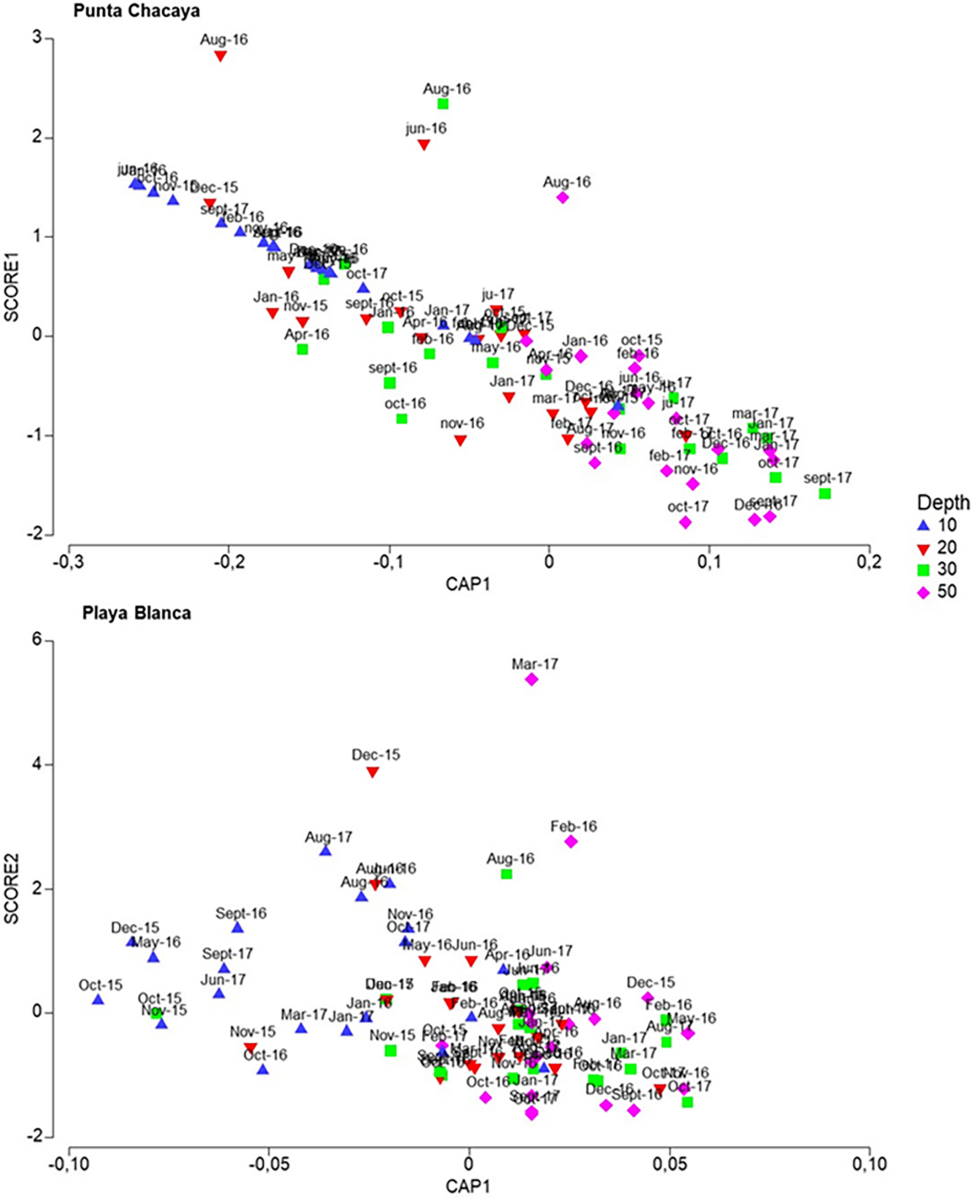
Relationships between community and environmental parameters. Draftsman plots showing the gradient generated between the community structure and environmental parameters.

At both sampling sites, PCA indicated that the first two principal components explained much of the environmental variability– 90.7% for Punta Chacaya and 69.6% for Playa Blanca. At both sites, dissolved oxygen, temperature, organic matter and to a lesser extent salinity scored the highest eigenvectors values for both principal components (Table 4). Biplots of canonical analyses of principal components show the environmental gradient at both sampling sites–warm, more oxygenated and high salinity values towards the shallow habitat, while more organic matter toward the deep habitat (Fig 9). The relationship between the CAP and PCA scores shows this gradient clearly (Fig 10).

**Table 4:** Results of principal component analysis (PCA). Largest eigenvectors are presented in bold.

	Punta Chacaya		Playa Blanca	
	PC1	PC2	PC1	PC2
**Eigenvalues**	1.53	0.25	2.5	1.54
**Proportion of variation**	77.9%	12.8%	43%	69.6%
**Cumulative variation**	77.9%	90.7%	43%	69.6%
**O**_**2**_ **ml/L**	**0.760**	-0.646	-0.475	0.238
**T°C**	**0.644**	0.752	**-0.621**	0.205
**Sal**	0.032	0.124	**0.574**	0.694
**O.M.**	-0.084	-0.036	0.244	**-0.648**

## Discussion

This study provides evidence in favor of the concept of macrobenthic community structure as a function of the environmental gradient produced by depth, bottom oxygenation, organic matter in sediment and bottom water temperature in a key upwelling region of northern Chile. Our results suggest a strong decrease on taxonomic richness and biomass as depth increases but an inverse pattern in abundance. The observed pattern in the community structure was correlated with the environmental gradient and throughout time despite the occurrence of oxygenation episodes. In contrast with our original hypothesis, during these oxygenation events, only subtle increments in taxonomic richness were detected in deep communities. An examination of the multivariable dispersion on community composition as a measure of beta diversity indicates that only very shallow communities are much more diverse and structured than those at depth, which is attributable to the compression effect generated by the semipermanent presence of a shallow hypoxic zone. In the context of the ongoing decline of dissolved oxygen in most oceanic and coastal regions and anthropogenic eutrophication [44]. It could be predicted that macrofaunal heterogeneity will decrease, thus, this parameter must be considered in monitoring programs of benthic habitats.

Previous studies on benthic communities in the Humboldt Current ecosystem have pointed to the occurrence of hypoxic episodes but in the form of pulse events. Hypoxia develops at moderated depths (ca. 100 m) then rises abruptly into shallow habitats, thus dramatically impacting the benthic community and often leading to mass mortality of several taxa [30]. In contrast, our results do not suggest a drastic impact during the occurrence of hypoxic waters in very shallow sediments. This may be due to the fact that, in our study location, shallow hypoxia was permanent almost year-round and thus resembled a situation much more akin to press disturbance. This finding indicates that the communities of at least 20 m depth and deeper are constantly disturbed by hypoxia. Under such conditions, the macrobenthos may experience hypoxia so frequently that a significant number of organisms may have adapted to this stressor. For example, polychaetes such as *Paraprionospio pinnata*, *Nephtys ferruginea*, *Glycera americana*, *Haploscoloplos* sp., *Lumbrineris composita*, *Sigambra bassi*, *Aricidea pigmentata*, *Cossura chilensis* and *Pectinaria chilensis* were prominent components of the communities at both sampling sites. These species present a suite of enzymes that facilitate anaerobic metabolism for coping with hypoxic conditions [45]. Particularly, the spionid polychaete *Paraprionospio pinnata* seems to be physiologically well-adapted for coping with conditions ranging from hypoxia to anoxia [46]. In our sampling, the polychaetes *Paraprionospio pinnata*, *Haploscoloplos* sp. and *Nephtys ferruginea* were commonly present along the seabed gradient. These three were present at all depths, though most abundant at 30 and 50 m, where oxygen concentrations ranged from 1 to 0.5 ml/l during most of the study period. Some species were found to be abundant at depths whose ambient environments fluctuated between normoxic and hypoxic conditions; *Glycera americana* and *Lumbrineris composite* were present at all depths, but more abundant at 10 and 20 m and at 20 and 30 m, respectively. At some extent, this may reflect different levels of physiological adaptation to the hypoxia gradient [31–33]. Indeed, throughout the Humboldt Current ecosystem, select species have demonstrated the ability to seek refuge in the upper layer of the oxygen minimum zone during ontogenic development, such as the case of the squat lobster *Pleuroncodes monodon* [47, 48]. At the Strait of Georgia, Vancouver Island, some epibenthic species (e.g., crustaceans) exposed to seasonal and severe hypoxia may persist, suggesting that region-specific hypoxia thresholds must be estimated to understand the impacts of future deoxygenation on marine biota [49].

Numerous studies have focused on documenting the impacts of strong pulses (seasonal or aseasonal) of hypoxic events and their effects on biota [28, 30]. However, dissolved oxygen profiles in this study depicted an inverse pattern; shallow hypoxia prevailed most of the time in the bay, and pulses of oxygenation occur in a seasonal basin. As mentioned earlier, oxygenation pulses did not cause enough of an impact to trigger a substantial recovery and development into a more mature and structured community [9]. Arguably, this may be because oxygenation episodes did not last long enough to produce such a switch in benthic, hypoxic communities. Indeed, throughout the latitudinal gradient of the Humboldt Current ecosystem, the responses of benthic communities to oxygenation pulses in northern Chile are quite different compared to the variability reported at northern and southern locations. In both central Peru (ca. 11°S) and central Chile (ca. 36°S), important differences in species richness, abundance, biomass and bioturbation capacity have been documented during the intrusion of warm, well-oxygenated and nutrient-poor waters during El Niño events [17–19]. In the coastal waters of Coloso (ca. 23°S in northern Chile), the assemblage of benthic polychaetes remained persistent without considerable changes during El Niño 97–97 [50, 51]. It is not clear why benthic communities in northern Chile do not present defined switches during seasonal oxygenation episodes, neither those detected during our monitoring nor during natural interannual variability such a ENSO events. Since upwelling is quite powerful and persistent at this latitude, this oceanographic process may be buffering any impact of episodic oxygenation events [52], at least short-term responses occurring at between months variability. Pacheco et al. [21] revealed that benthic communities at Coloso indeed did not show an immediate response to the ENSO event, but rather reveal a pattern of gradual changes in community structure between years before El Niño 97–98 and a similar pattern post La Niña 99–00, resembling a decadal cycle. Therefore, short-term responses may not occur throughout the northern Chile ecosystem.

Our study and the contrast with other pieces of evidence throughout the Humboldt Current system suggest that understanding benthic community responses to oscillating normoxic to hypoxic conditions requires region-specific monitoring. This becomes more relevant in the context of the current and projected trends of dissolved oxygen declines in most ocean and coastal regions [44]. Upwelling systems are particularly susceptible to deoxygenation because warming reinforces trade winds, therefore making upwelling more intense [14, 15]. Upwelling transports subsurface, oxygen-poor waters that would typically stay on the shelf for prolonged time periods to the surface. It could be predicted that in such a scenario, oxygenation episodes would not persist long enough for communities recovering from hypoxic conditions, similarly to what we show in our data set. As suggested by Breitburg et al. [44], monitoring of oxygen variability in coastal areas should be implemented to detect and direct management of the occurrence and extent of deoxygenation events or improvements in oxygen conditions.

We have used beta diversity (i.e. variation of taxa over an environmental gradient) to measure the multivariable dispersion of community composition (presence/absence of taxa). Beta diversity changed along the environmental gradient and the variability of the community composition significantly decreased from shallow normoxic to deep hypoxic sediments. Intense changes in beta diversity occur in areas with strong environmental filtering, such as the boundaries of biogeographic regions where community composition is the result of different levels of tolerance to environmental conditions or disturbances. Our results indicate that communities not only lack species richness (alpha diversity), but also community heterogeneity is reduced, likely explained by the filtering effect of hypoxic conditions. Similar results have been reported for ground fish assemblages within the California current system. Beta diversity decreased with depth but peaked in the transitions between the upper and lower border of the oxygen minimum zone in that ecosystem [53]. On the Oman margin, polychaete assemblages within the oxygen minimum shown a considerable reduced multivariate dispersion in comparison to those below the OMZ [54]. We suggest that beta diversity measurements could be implemented in studies and monitoring programs of communities’ subject to hypoxia.

## Supporting information

S1 Table Appendix. Community data.
(XLSX)

## References

[1] Chavez FP, Messie M. A comparison of eastern boundary upwelling ecosystems. Prog Oceanogr. 2009;83: 80–96. 10.1016/j.pocean.2009.07.032

[2] Levin LA. Oxygen minimun zone benthos: adaptation and community response to hypoxia. Oceanogr Mar Biol Ann Rev. 2003;41: 1–45.

[3] Rosenberg R, Arntz WE, Chumán de Flores E, Flores LA, Carbajal G, Finger I, et al Benthos biomass and oxygen deficiency in the upwelling system off Peru. J Mar Res. 1983; 41: 263–279. 10.1357/002224083788520153

[4] Arntz WE, Tarazona J, Gallardo VA, Flores LA, Salzwedel H. Benthos communities in oxygen deficient shelf and upper slope areas of the Peruvian and Chilean Pacific coast, and changes caused by El Niño In: Tyson RV, Pearson TH, editors. Modern and ancient continental shelf anoxia. Geol Soc Spec Publ; 1991 no. 58, pp. 131–154.

[5] Arntz W, Gallardo V, Gutiérrez D, Isla E, Levin L, Mendo J, et al El Niño and similar perturbation effects on the benthos of the Humboldt, California and Benguela current upwelling ecosystems. Adv Geosci. 2006; 6:243–265. 10.5194/adgeo-6-243-2006

[6] Quiroga E, Quiñones R, Palma M, Sellanes J, Gallardo VA, Gerdes D, et al Biomass size-spectra of macrobenthic communities in the oxygen minimum zone off Chile. Estuar Coast Shelf Sci. 2005;62: 217–231. 10.1016/j.ecss.2004.08.020

[7] Palma M, Quiroga E, Gallardo V, Arntz W, Gerdes D, Schneider W, et al Macrobenthic animal assemblages of continental margin off Chile (22° to 42°S). J Mar Biol Ass UK. 2005;85: 233–245. 10.1017/S0025315405011112h

[8] Pacheco AS, Gonzales MT, Bremner J, Laudien J, Oliva M, Heilmayer O, et al Functional diversity of marine macrobenthic communities from sublittoral soft-bottom habitats off northern Chile. Helgol Mar Res. 2011;65: 413–424. 10.1007/s10152-010-0238-8

[9] Díaz RJ, Rosenberg R. Spreading dead zones and consequences for marine ecosystems. Science. 2008; 321: 926–929. 10.1126/science.1156401 18703733

[10] Díaz RJ, Rosenberg R, Rabalais NN, Levin LA. Dead zone dilemma. Mar Poll Bull 2009;58: 1767–1768. 10.1016/j.marpolbul.2009.09.030 19853871

[11] Keeling RF, Körtzinger A, Gruber N. Ocean deoxygenation in a warming world. Annu Rev Mar Sci. 2010;2: 199–229. 10.1146/annurev.marine.010908.163855 21141663

[12] Schmidtko S, Stramma L, Visbeck M. Decline in global oceanic oxygen content during the past five decades. Nature. 2017; 542: 335–542. 10.1038/nature21399 28202958

[13] Stramma L, Schmidtko S, Levin LA, Johnson GC. Ocean oxygen minima expansions and their biological impacts. Deep Sea Res Part 1. 2010;57: 587–595. 10.1016/j.dsr.2010.01.005

[14] Gutiérrez D, Bouloubassi I, Sifeddine A, Purca S, Goubanova K, Graco M, et al Coastal cooling and increased productivity in the main upwelling zone off Peru since the mid twentieth century. Geophys Res Lett. 2011;38: L07603, 10.1029/2010GL046324

[15] Schneider W, Donoso D, Garcés-Vargas J, Escribano R. Water-column cooling and sea surface salinity increase in the upwelling region off central-south Chile driven by a poleward displacement of the South Pacific High. Prog Oceanogr. 2017;151: 38–48. 10.1016/j.pocean.2016.11.004

[16] Chan F, Barth JA, Lubchenco J, Kirincich A, Weeks H, Peterson WT, et al Emergence of anoxia in the California current large marine ecosystem. Science. 2008;319: 920 10.1126/science.1149016 18276882

[17] Tarazona J, Arntz WE, Canahuire E. Impact of two ‘‘El Niño” events of different intensity on the hypoxic soft bottom macrozoobenthos off the central Peruvian coast. PSZN Mar Ecol. 1996;17: 425–446. 10.1111/j.1439-0485.1996.tb00519.x

[18] Sellanes J, Quiroga E, Neira C, Gutiérrez D. Changes of macrobentos composition under different ENSO cycle conditions on the continental shelf off central Chile. Cont Shel Res 2007; 27: 1002–1016. 10.1016/j.csr.2007.01.001

[19] Gutiérrez D, Gallardo VA, Mayor S, Neira C, Vásquez C, Sellanes J, et al Effects of dissolved oxygen and fresh organic matter on the bioturbation potential of macrofauna in sublittoral sediments off Central Chile during the 1997/1998 El Niño. Mar Ecol Prog Ser. 2000;202: 81–99. 10.3354/meps202081

[20] Gutiérrez D, Enríquez E, Purca S, Quipúzcoa L, Marquina R, Flores G, et al Oxygenation episodes on the continental shelf of Peru: remote forcing and benthic ecosystem response. Prog Oceanogr. 2008;79: 177–189. 10.1016/j.pocean.2008.10.025

[21] Pacheco AS, Riascos JM, Orellana F, Oliva ME. El Niño-Southern Oscillation cyclical modulation of macrobenthic community structure in the Humboldt Current ecosystem. Oikos 2012;121: 2097–2109. 10.1111/j.1600-0706.2012.20257.x

[22] Underwood AJ. Importance of experimental design in detecting and measuring stresses in marine populations. J Aquat Ecosystem Stress and Recovery. 2000; 7: 3–24. 10.1023/A:1009983229076

[23] Fleddum A, Cheung SG, Hodgson P, Shin PKS. Impact of hypoxia on the structure and function of benthic epifauna in Tolo Harbour, Hong Kong. Mar Poll Bull. 2011; 63: 221–229. 10.1016/j.marpolbul.2011.03.019 21481897

[24] Froehlich HE, Hennessey SM, Essington TE, Beaudreau AH, Levin PS. Spatial and temporal variation in nearshore macrofaunal community structure in a seasonally hypoxic estuary. Mar Ecol Prog Ser 2015; 520:67–83. 10.3354/meps11105

[25] Tweedley JR, Hallett CS, Warwick RM, Clarke RK, Potter IC. The hypoxia that developed in a microtidal estuary following an extreme storm produced dramatic changes in the benthos. Mar Fresh Res 2015;67: 327–341. 10.1071/MF14216

[26] Gammal J, Norkko J, Pilditch CA, Norkko A. Coastal hypoxia and the importance of benthic macrofaunal communities for ecosystem functioning. Estuaries and Coasts 2016; 40: 457–468. 10.1007/s12237-016-0152-7

[27] Rakocinski CF, Menke DP. Seasonal hypoxia regulates macrobenthic function and structure in the Mississipi Bight. Mar Poll Bull 2016;105: 200–309. 10.1016/j.marpolbul.2016.02.006 26920427

[28] Veas R, Hernández-Miranda E, Quiñones R, Carrasco FD. Spatio-temporal biodiversity of soft bottom macrofaunal assemblages in shallow coastal waters exposed to episodic hypoxic events. Mar Environ Res. 2012;78: 1–14. 10.1016/j.marenvres.2012.02.008 22521572

[29] Soto E, Quiroga E, Ganga B, Alarcon G. Influence of organic matter inputs and grain size of soft-bottom macrobenthic biodiversity in the upwelling ecosystem of central Chile. Mar Biodivers 2016; 10.1007/s12526-016-0479-0

[30] Hernandez-Miranda E, Veas R, Labra FA, Salamanca M, Quiñones RA. Response of the epibenthic macrofaunal community to a strong upwelling-driven hypoxic event in a shallow bay of the southern Humboldt Current System. Mar Environ Res. 2012; 79: 16–28. 10.1016/j.marenvres.2012.04.004 22626877

[31] Diaz RJ, Rosenberg R. Marine benthic hypoxia: a review of its ecological effects and the behavioral responses of benthic macrofaunal. Oceanogr Mar Biol Ann Rev. 1995; 33: 245–303.

[32] Gray JS, Wu RSS, Or YY. Effects of hypoxia and organic enrichment on the coastal marine environment. Mar Ecol Prog Ser. 2002; 238:249–279. 10.3354/meps238249

[33] Vaquer-Sunyer R, Duarte CM. Thresholds of hypoxia for marine biodiversity. Proc Natl Acad Sci USA 2008;105: 15452–15457. 10.1073/pnas.0803833105 18824689 PMC2556360

[34] IFOP. Monitoreo de las condiciones bio-oceanográficas entre la XV y IV Regiones, año 2009. FIP N° 2009–38. diciembre 2010. http://www.fip.cl/Archivos/Documentacion/Noticias/boletin%20N%2011%20Agosto%202010.pdf

[35] Marín V, Escribano R, Delgado LE, Olivares G, Hidalgo P. Nearshore circulation in a coastal upwelling site off the Northern Humboldt current system. Cont Shelf Res. 2001;21, 1317–1329. 10.1016/S0278-4343(01)00022-X

[36] Laudien J, Rojo M, Oliva M, Arntz W, Thatje S. Sublittoral soft bottom communities and diversity of Mejillones Bay in northern Chile (Humboldt Current upwelling system). Helgoland Mar Res 2007;61: 103–116. 10.1007/s10152-007-0057-8

[37] Guiñez M, Valdés J, Sifeddine A. Variabilidad espacial y temporal de la materia orgánica sedimentaria, asociada a la Zona de Mínimo Oxígeno (ZMO), en un ambiente costero del norte de la corriente de Humboldt, bahía de Mejillones, Chile. Lat Am J Aquat Res. 2010; 38: 242–253 10.3856/vol38-issue2-fulltext-9

[38] Clarke KR Non-parametric multivariate analyses of changes in community structure. Australian J Ecol. 1993; 18:117–143. 10.1111/j.1442-9993.1993.tb00438.x

[39] Anderson M.J. (2001) A new method for non-parametric multivariate analysis of variance. Austral Ecol. 26, 32–46. 10.1111/j.1442-9993.2001.01070.pp.x

[40] Clarke KR, Warwick RM. Quantifying structural redundancy in ecological communities. Oecologia 1998; 113: 278–289 10.1007/s004420050379 28308208

[41] Anderson MJ, Crist TO, Chase JM, Vellend M, Inouye BD, Freestone Al et al Navigating the multiple meanings of B diversity: a roadmap for practicing ecologist. Ecol Lett. 2011; 14: 19–28 10.1111/j.1461-0248.2010.01552.x 21070562

[42] Anderson MJ, Ellingsen KE, McArdle BH. Multivariate dispersion as a measure of beta diversity. Ecol Lett. 2006; 9: 683–693. 10.1111/j.1461-0248.2006.00926.x 16706913

[43] Anderson MJ, Gorley RN, Clarke KR. PERMANOVA+ for PRIMER: Guide to software and statistical methods PRIMER-E: Plymouth, UK 2008

[44] Breitburg D, Levin LA, Oschlies A, Grégoire M, Chavez FP, Conley DJ, et al Declining oxygen in the global ocean and coastal waters. Science 2018; 359, eaam7240. 10.1126/science.aam7240 29301986

[45] González RR, Quiñones RA. Pyruvate oxidoreductases involved in glycolytic anaerobic metabolism of polychaetes from the continental shelf off central-south Chile. Est Cont Shelf Res. 2000; 51: 507–519 10.1006/ecss.2000.0693

[46] Quiroga E, Quiñones RA, González RR, Gallardo VA. Aerobic and anaerobic metabolism of *Paraprionospio pinnata* (Polychaeta: Spionidae) in central Chile. J Mar Biol Ass UK. 2007; 87:459–463. 10.1017/S0025315407048710

[47] Yannicelli B, Paschke K, González RR, Castro LR. Metabolic responses of the squat lobster (*Pleuroncodes monodon*) larvae to low oxygen concentration. Mar Biol. 2013; 160:961–976. 10.1007/s00227-012-2147-7

[48] Gallardo MA, González Lopéz AE, Ramos M, Mujica A, Muñoz P, Sellanes P, Yannicelli B. Reproductive patterns in demersal crustaceans from the upper boundary of the OMZ off north-central Chile. Cont Shel Res. 2017; 141:26–37 10.1016/j.csr.2017.04.011

[49] Chu JWF, Tunnicliffe V. Oxygen limitations on marine animal distribution and the collapse of epibenthic community structure during shoaling hypoxia. Global Change Biol. 2015; 21: 2989–3004. 10.1111/gcb.12898 25689932

[50] Carrasco FD. Sublittoral macrobenthic fauna off Punta Coloso, Antofagasta, northern Chile: high persistence of the polychaete assemblage. Bull Mar Sci 1997; 60: 443–459.

[51] Carrasco FD, Moreno RA. Long-term dynamics (1990 to 2004) of the polychaete fauna from the sublittoral soft-bottoms off Punta Coloso (Antofagasta), northern Chile. Sci Mar 2006; 70S3: 169–178.

[52] Camus PA. Understanding biological impacts of ENSO on the eastern Pacific: an evolving scenario. Int J Environ Health 2008; 2:5–19. 10.1504/IJEnvH.2008.018668

[53] Anderson MJ, Tolimieri N, Millar RB. Beta diversity of demersal fish assemblages in the north-eastern Pacific: interactions of latitude and depth. PLoS ONE 2013; 8(3): e57918 10.1371/journal.pone.0057918 23526960 PMC3602450

[54] Levin LA, Gage JD, Martin C, Lamont PA. Macrobenthic community structure within and beneath the oxygen minimum zone, NW Arabian Sea. Deep-Sea Res PTII 2000; 47:189–226. 10.1016/S0967-0645(99)00103-4

